# Accuracy of breeding values of 'unrelated' individuals predicted by dense SNP genotyping

**DOI:** 10.1186/1297-9686-41-35

**Published:** 2009-06-11

**Authors:** Theo HE Meuwissen

**Affiliations:** 1 Department of Animal and Aquacultural Sciences, Norwegian University of Life Sciences, Box 1432, Ås, Norway

## Abstract

**Background:**

Recent developments in SNP discovery and high throughput genotyping technology have made the use of high-density SNP markers to predict breeding values feasible. This involves estimation of the SNP effects in a training data set, and use of these estimates to evaluate the breeding values of other 'evaluation' individuals. Simulation studies have shown that these predictions of breeding values can be accurate, when training and evaluation individuals are (closely) related. However, many general applications of genomic selection require the prediction of breeding values of 'unrelated' individuals, i.e. individuals from the same population, but not particularly closely related to the training individuals.

**Methods:**

Accuracy of selection was investigated by computer simulation of small populations. Using scaling arguments, the results were extended to different populations, training data sets and genome sizes, and different trait heritabilities.

**Results:**

Prediction of breeding values of unrelated individuals required a substantially higher marker density and number of training records than when prediction individuals were offspring of training individuals. However, when the number of records was 2*N_e_*L and the number of markers was 10*N_e_*L, the breeding values of unrelated individuals could be predicted with accuracies of 0.88 – 0.93, where N_e _is the effective population size and L the genome size in Morgan. Reducing this requirement to 1*N_e_*L individuals, reduced prediction accuracies to 0.73–0.83.

**Conclusion:**

For livestock populations, 1N_e_L requires about ~30,000 training records, but this may be reduced if training and evaluation animals are related. A prediction equation is presented, that predicts accuracy when training and evaluation individuals are related. For humans, 1N_e_L requires ~350,000 individuals, which means that human disease risk prediction is possible only for diseases that are determined by a limited number of genes. Otherwise, genotyping and phenotypic recording need to become very common in the future.

## Background

The Human Genome Project and related projects for other species have generated the complete DNA sequence of many animal, plant, and microbial genomes . An important result from these sequencing efforts is the detection of 100,000's to millions of SNP markers for each of the sequenced species. The availability of all these SNP and recent innovations in high-throughput SNP-chip genotyping technology have made the routine genotyping of huge SNP panels feasible. For example, in human genetics, assays with > 500,000 SNP are routinely used, and in cattle, pigs and sheep ~50,000 SNP chips are commercially available.

These dense marker genotypes can be used to predict genome-wide breeding values using genomic selection (e.g. [1,2]). Genomic selection consists of the following steps: (i) estimation of the effects of all markers in a 'training data set', where the individuals are phenotyped and genotyped; (ii) prediction of the breeding values of other 'evaluation' individuals by combining their marker genotypes with the estimates obtained in step (i). These steps implicitly assume that there is substantial linkage disequilibrium (LD) between the markers and the QTL, and, ideally, for every QTL there is a marker in perfect LD, i.e. R^2 ^= 1, where R^2 ^is the square of the correlation between the allele frequencies at two loci. Habier *et al*. [3] have demonstrated that breeding values can also be predicted in the absence of linkage between markers and QTL, since the markers can predict family relationships between the individuals. However, substantial LD requires strong linkage, especially for the prediction of unrelated individuals, and thus dense marker genotyping.

The ideal of having a marker in perfect LD with each QTL is complicated by the fact that recently, it has been shown in human genetics studies, that nearly all the genetic variation of quantitative traits is due to genes with a small effect [4]. This implies that (i) there are very many QTL, and thus that the effect of a single marker may be due to a number of QTL in the region; (ii) the estimation of single gene effects will be complicated by their small size and LD with other genes; (iii) assuming a constant genetic variance across the genome when estimating marker effects may be quite realistic, as was shown by Visscher *et al*. [5] for height in humans. The latter favours the BLUP model for the estimation of marker effects relative to non-linear models, which give more weight to positions that appear to have large effects (e.g. the BayesB model [1]).

In the step estimating marker effects, the estimation of effects of very many markers is hampered by the LD, i.e. collinearity, between the marker effects. Fortunately, similar combinations of marker alleles will be found in the evaluation data set (step (ii)), especially if the individuals of steps (i) and (ii) are related (e.g. parents and offspring as in [2]). The latter implies that it is not necessary to estimate the effect of single markers accurately, as long as the effects of distinct haplotypes are estimated accurately by summing the effects of their marker alleles. The prediction of breeding values of 'unrelated' individuals is a particularly poor case, since the haplotypes in the evaluation data set can be very different from those in the training data set. Here, 'unrelated' individuals means that they are from the same population, but not structurally related to the training data individuals. However, the prediction of breeding values of unrelated individuals is exactly what is required in many and perhaps the most promising applications of genomic selection, for example when using field data to predict breeding values of elite breeding stocks, the selection of individuals for markers whose effects were estimated in an experiment on a unrelated subset of individuals, and in the case of genetic risk prediction for human diseases [6].

The aim of this study is to assess whether the breeding values of unrelated individuals could be predicted with high accuracy, and what resources are required in terms of marker density and number of records in the training data set. The results are based on computer simulations of relatively small populations, but will be generalised using the scaling by effective size (N_e_) argument from coalescence theory [7,8].

## Methods

### The scaling by N_e _argument

From the coalescence theory it is well known that, for a population in recombination-drift equilibrium, the LD between marker and QTL and amongst markers is a function of N_e_*c, where c is the recombination rate between the loci and N_e _is the effective population size. For instance, the LD structure will be the same for a population with N_e _= 100 and 1000 SNPs per Morgan (M), compared to a population with N_e _= 1000 and 10,000 SNPs per M, i.e. for both populations, the marker density is 10 * N_e _/M [9].

However, the second population requires the estimation of 10 times as many markers, which may be achieved with a similar accuracy if we have 10 times as many training data. The latter is also seen from recent predictions of the accuracy of selection [10-12]:

(1)

where *r *is accuracy of selection; N is the number of phenotypic training records; h^2 ^is the trait heritability; L is genome size in Morgan; 4 *N*_e_*Lν *is the effective number of QTL loci in the genome, which each equals the effective number of segments in the genome when the infinitesimal model is assumed (*i.e*. BLUP is used for the estimation of SNP effects). In the latter case, ν may be interpreted as the ratio of the effective number of segments and the actual number of segments, which is expected to be 4 *N*_e_*L*. Goddard [11] derived that the effective number of segments is , where summation is over the chromosomes and L_i _is the size of chromosome i.

From this scaling argument and Equation (1) it is also seen that as genome size doubles, we need twice as many training records (N) to achieve a similar accuracy of predicting breeding value, assuming a constant marker density. Whether the latter expectation holds will be tested in the Results and Discussion section. Also, the LD structure between the QTL is equivalent if the number of QTL per M is 100 and 1000 in populations with N_e _= 100 and 1000, respectively.

In order to reduce computer time, the effective size used in the simulations described here will be quite low (N_e _= 100), but the scaling argument makes it possible to extend the results to bigger population sizes. The use of a relatively low N_e _does not only reduce the population size to be simulated, but also the number of generations needed to reach equilibrium between mutation, drift and recombination. This is because lineages coalesce faster in small populations.

### The genomic history of the populations

In general, the model for the population history mimics that of coalescence simulations [7], however a forward simulation approach is used because this increases the size of the chromosomes that can be handled. Following the coalescence theory, the Fisher-Wright idealised population model [13] and the infinite-sites mutation model were assumed [14], with a mutation frequency of 2*10^-8 ^per nucleotide per generation. The latter ensured a large number of SNP. The historical effective size of the population was N_e _= 100, and the forward simulations were conducted for 400 generations. The latter is expected to result in a mutation-drift balance, since any sample of alleles at a locus is expected to coalesce into its most recent common ancestor (MRCA) within 200 (= 2N_e_) generations. Any mutations before this MRCA lived do not cause a polymorphism (since all present alleles would be of the mutant type). Preliminary simulations showed that an approximate mutation-drift balance was reached before 400 generations (result not shown). Recombinations were sampled according to the Haldane mapping function. The genome consisted of 10 chromosomes of 50 cM each, i.e. the total genome size was 5 M.

After these 400 generations, SNP with a Minor Allele Frequency (MAF) < 0.02 were discarded. From the remaining SNP, 12 were randomly selected per chromosome to become a QTL, which resulted in a total of 120 QTL. From the remaining, non-QTL SNP, the 1000 SNP per chromosome with the highest MAF were selected to become a marker. This resulted in a total of 10,000 markers, and a density of 20 N_e_/M. For humans, this density corresponds to a total of ~2.3 million markers (= 20*38*3,000; assuming a genome of 38 M [15], and N_e _~3,000 [16]), and for cattle 600,000 markers (assuming a 30 M genome and N_e_~1,000). Smaller marker densities of 20/x N_e_/M were obtained by taking every x-th marker from the original set of 10,000 markers, where x = 2, 4, 10 or 20.

### Recent history of the populations

**Figure 1:**
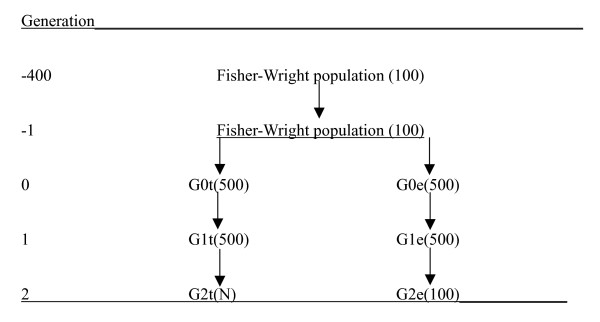
**A schematic representation of the population structure**. (population sizes are indicated between brackets).

After these 400 generations, the population was increased to 1000 by sampling parents from the previous generations for 1000 individuals, which formed generation G0. Generation G0 was split into 500 G0t and 500 G0e individuals (e and t indicate that they become the 'evaluation' and 'training' line, respectively). The 500 G0e-individuals were used for the sampling of parents for 500 G1e individuals, and similarly the G0t-individuals were used for the sampling of parents for 500 G1t individuals. Setting up different lines for the sampling of the G1e and the G1t individuals ensured that these two groups of individuals shared no close relationships. Subsequently, parents of 100 G2e individuals were sampled at random (with replacement) from the 500 G1e individuals. Similarly, parents of N G2t individuals were sampled from the 500 G1t individuals, where N was 500, 1000 or 2000. The G2t individuals were used for the estimation of marker effects, *i.e*. they were genotyped and phenotyped. The 100 G2e individuals are only genotyped, and their genetic value is to be predicted. The G0e, G0t, G1e, G1t, G2e and G2t individuals were pedigree recorded, i.e. for the pedigree recording the parents of G0 were treated as founders. The 'training' individuals (G2t) had neither parents nor grandparents in common with the evaluation individuals (G2e) due to the separation of the two lines. The results were based on 16 replicated simulations, which was computationally advantageous, since the 16 replicates could be run in parallel. Figure 1 summarises the population structure.

### Genetic and phenotypic values

An additive genetic model was assumed, and the allelic effect of the mutant QTL allele at locus j, u_j_, was sampled from the exponential distribution, and u_j _was given a negative sign with probability 0.5. The total genetic value of individual i was calculated as:



where q_ij _is the number of mutant alleles (0, 1, or 2) that individual i carries at locus j. At the end of the 400 generations of simulation, the allelic effects were standardised so that the total genetic variance was 1. Phenotypes for the G2t individuals were obtained by adding an environmental effect sampled from N(0,0.25) to their genetic value. This resulted in a high heritability of 0.8. The effect of a lower heritability is investigated in the Results and Discussion section.

### Estimation of marker effects and prediction of breeding value

Estimation of marker effects was performed using three models: (i) BLUP of marker effects, which assumes that every marker effect has a constant variance (G-BLUP); (ii) BayesB, which estimates the variance of every marker using a prior distribution and Bayesian methodology [2]; and (iii) MIXTURE, which assumes that the marker effects come from a mixture of two normal distributions, which differ in variance, i.e. the large marker effects are accommodated by the distribution with large variance and vice versa. The MIXTURE model was used because it, in a relatively simple way, approximates the prior distribution of the marker effects, assuming that any prior distribution can be reasonably well approximated by a mixture of normal distributions [17]. Some preliminary testing of the MIXTURE model showed that a mixture of two normal distributions is sufficient. The prior distribution of BayesB of [2] assumed that some markers had a big effect, the variance of which was estimated (a fraction N_QTL_/N_m _of markers), whilst the remaining markers did not have an effect at all, where N_QTL _is the number of QTL and N_m _is the number of markers fitted. However, in the BayesB model implemented here, the prior distribution assumed that the majority of the markers (i.e. the fraction 1- N_QTL_/N_m_) did have a small effect, the variance of which was assumed equal and was estimated in the model, instead of assuming that these markers had no effect at all (as in [2]). The latter has two advantages: (i) a Gibbs-sampling algorithm can be implemented, which reduces computer time; and (ii) since there were many QTL, they will probably not be all clearly detected by a single marker, such that a proportion of the genetic variance will be picked up by allowing for many, small marker effects.

The statistical model used to estimate the marker effects by G-BLUP, BayesB, and MIXTURE was:



where **y **is a Nx1 vector of phenotypes; a_j _is the effect of marker j; **X**_**j **_is a Nx1 vector denoting the genotype of the individuals for marker j, where 0 denotes homozygous for the first allele; 1/√H_j _denotes heterozygous; 2/√H_j _denotes homozygous for the second allele, and H_j _is the marker heterozygosity. The √H_j _term in **X**_**j **_standardises the variance of the marker genotype data to 1. The variance of a_j _is assumed to be 1/N_m _for G-BLUP, is estimated by BayesB, and, in MIXTURE it equals σ_1_^2 ^or σ_2_^2^, depending on whether the marker effect is small or large. The probability of a small or large marker effect is estimated together with the variances of the small and large distribution of marker effects, σ_1_^2 ^and σ_2_^2^.

Given the estimates of the marker effects and the marker genotypes, genetic values for the individuals in set G2e are predicted as:



where X_ij _is the marker genotype of individual i for marker j coded the same as above; and  is the estimate of marker effect j. The accuracy of this prediction is calculated as the correlation between *g*_i _and  for the G2e individuals.

Traditional BLUP (T-BLUP [18]) breeding values are estimated based on the phenotypes of the individuals in G2t and the pedigree of the G0, G1t, G1e, G2t, and G2e individuals using the ASREML package [19].

### Testing the effect of an increase in genome size

From Equation (1) it may be expected that a doubling of the genome size requires twice as many records. To test this expectation, we compared the situation of a genome with 5 chromosomes with N = 500 G2t individuals, to 10 chromosomes with N = 1000, and to 20 chromosomes with N = 2000. Marker density was kept constant at 20 N_e_/M.

### Accounting for relationships

Following Habier *et al*. [3], the accuracy of G-BLUP may be split into a component due to genomic selection and a component that could also be predicted by T-BLUP, i.e.:

(2)

where r_G-BLUP _(r_T-BLUP_) is the accuracy using G-BLUP (T-BLUP), (1-r_T-BLUP_) denotes the inaccuracy of r_G-BLUP_, and ρ_G-BLUP _is the proportion of the inaccuracy that could be explained by G-BLUP. Since, r_G-BLUP _and r_T-BLUP _are known, ρ_G-BLUP_can be calculated from the simulation results and Equation (2). Using these ρ_G-BLUP _values, a method to predict the accuracies from traditional BLUP (e.g. [20]), and Equation (2), we can predict r_G-BLUP _in situations where there may be very different relationships between the training and evaluation individuals, than assumed in the presented simulations. Similarly, the accuracy of BayesB can be predicted for different relationships between training and evaluation individuals.

To test these predictions, a simulation was conducted where the training data set was composed of G1t individuals, the number of which was increased to N, and the evaluation individuals originated from G1e. Hence, the training and evaluation individuals were two generations less separated (one generation in each of the lines).

### Estimation of the effective number of segments

By combining the simulation results and Equation (1), the effective number of segments can be derived as follows. Let PEV = 1-r_G-BLUP_^2^, then it can be seen from Equation (1) that the E(PEV^-1^) = 1+β*N, where . Hence, the regression of PEV^-1 ^on N is linear, and the regression coefficient is a function of h^2^, which is known, and the effective number of segments, 4N_e_Lν.

## Results and discussion

### Effect of number of markers and training records

**Figure 2:**
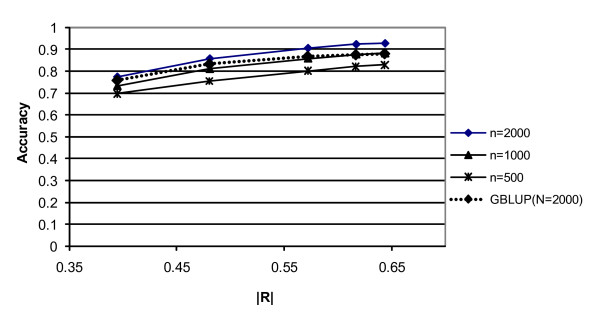
**Accuracy of the prediction of genetic values of G2e individuals for BayesB (except for the dashed line which indicates GBLUP) as a function of the marker density, which is expressed as the square root of R^2 ^between adjacent markers**. The markers shown from left to right are at densities of 1, 2, 5, and 10 Ne/M.

Figure 2 shows the accuracy of the predicted breeding values of the G2e individuals, as a function of the marker density expressed in terms of the linkage disequilibrium between adjacent markers (following Calus *et al*. [21]). The linkage disequilibrium between adjacent markers was calculated as R^2 ^= 1/(4N_e_d+1) [22], where d is the distance between the adjacent markers. As can be seen from Figure 2, accuracy increases approximately linearly with |R| over a 20-fold increase in marker density. However, increasing the density from 10 to 20 N_e_/M hardly increased the accuracy of selection (and also the |R| between adjacent markers). For G-BLUP, the slope of the increase with increasing density was clearly smaller, which indicates that the superiority of BayesB increases with increasing density. This may be expected since with increasing density it becomes more important to filter the SNP that are in strong LD with the QTL from all the others, instead of spreading the effects over all SNP as G-BLUP does, which results in very small effects for the single SNP.

The differences between BayesB and MIXTURE are very small (Table 1), but slightly in favour of BayesB, which is probably due to the informative prior distribution that was used in BayesB. G-BLUP yielded clearly lower accuracy at high density, which was especially the case for low N. This may be explained by the fact that as N goes to infinity all methods will reach perfect predictions of SNP effects, as can be seen from Equation (1). At low density (1 N_e_/M) G-BLUP yielded only a 0.02–0.06 fold lower accuracy than BayesB.

**Table 1:** A comparison of the accuracy of genetic value prediction of G2e individuals between BayesB and MIXTURE for extreme sizes of the training data set (N) and the marker density

**N**	**Marker density (N_e_/M)**	**BayesB**	**MIXTURE**	**G-BLUP**
2000	20	0.928	0.925	0.881
	1	0.773	0.772	0.758
1000	20	0.882	0.880	0.817
	1	0.732	0.735	0.717
500	20	0.829	0.825	0.727
	1	0.697	0.700	0.657

For T-BLUP, the accuracies varied between 0.19 and 0.23 as N increased from 500 to 2000 (result not shown elsewhere). Thus T-BLUP was much less accurate than BayesB (varied from 0.83 – 0.93) because (i) it does not make use of the marker data; and (ii) it uses pedigree-based relationships to predict the EBV of the evaluation individuals from the phenotyped of the training individuals which were generally low, on average 0.01.

**Figure 3:**
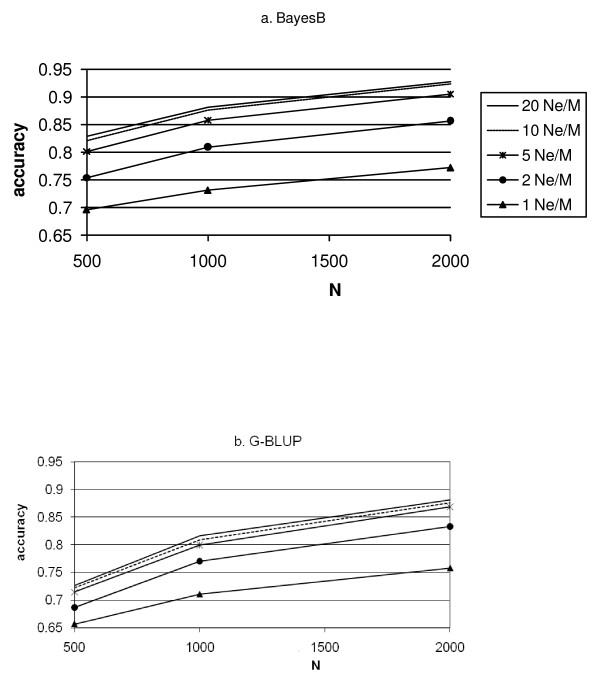
**Accuracy of the prediction of genetic values of G2e individuals for BayesB (3a) and G-BLUP (3b) as a function of the number of records in the training data set (N)**.

The accuracy of G-BLUP increases more than that of BayesB when the number of records increases (Figure 3). Thus, G-BLUP requires more records, N, to achieve high accuracy than BayesB. In other words, BayesB seems especially superior to G-BLUP in situations with small numbers of records and high marker density. In these situations, the prior knowledge about QTL effects used by BayesB partly overcomes the low information content of the data, and the high marker density results in marker effects reflecting better the effects of QTL. The effect of increasing the number of records, N, is smaller at low marker densities, especially for G-BLUP at density 1 Ne/M. For high densities, the accuracy keeps increasing as the number of records increases. Hence, to take advantage of high-density SNP genotyping, large data sets are needed to estimate the marker effects.

### Larger genome sizes

Table 2 shows the effect of doubling the genome size and simultaneously doubling the number of G2t individuals (N). From Equation (1), it was expected that accuracy would not be affected by this doubling. This is approximately the case, but not quite: for G-BLUP the accuracy decreases on average by 0.014 per doubling of genome sizes and for BayesB this figure is on average 0.0075. The mechanics of doubling genome size and numbers of training records may become clearer, if we consider two replicated simulations containing one chromosome each, and obtain an average accuracy of r. Now, if we combine the two chromosomes of the two replicates into one replicate with two chromosomes, it becomes clear that we also have to combine the phenotypic recordings of both replicates to predict marker effects and thus breeding values with the same accuracy. However, 2N records on two chromosomes are only as informative as N records on 1 chromosome, if the markers on chromosome 1 are independent to those of chromosome 2 (i.e. a balanced design). The markers on the two chromosomes are independent, but the number of markers is so large that some confounding between the markers of the two chromosomes will still occur by chance. The latter probably resulted in the somewhat reduced accuracy when doubling the genome size and the number of phenotypes.

**Table 2:** Effect of doubling simultaneously the genome size and the size of the training data set (N) on the accuracy of predictions of genetic values using a marker density of 20 Ne/M

**Genome size (M)**	**N**	**BayesB**	**G-BLUP**
2.5	500	0.890	0.831
5	1000	0.882	0.817
10	2000	0.875	0.803

### Accounting for relationships

Table 3 shows the errors of the predictions from Equation (2), when G1t was used as a training and G1e as an estimation data set, using BayesB and G-BLUP and the extremes of the marker densities. The accuracy of T-BLUP increased to 0.342, 0.410, and 0.412, for N = 500, 1000 and 2000, respectively, for these data sets, which was used in the prediction Equation (2). The errors of the predicted accuracies were all smaller than 0.027, and may in part be due to sampling errors from the Monte Carlo simulations. In general, it seems that Equation (2) provides quite precise predictions of the accuracies for different degrees of relationship between the evaluation and training individuals.

**Table 3:** Errors of predicting accuracies by Equation (1) (rEqn(1)) relative to simulation results (rsim), when G1t was used as training data set and the genetic values of G1e individuals were predicted

		**r_sim_-r_Eqn (1)_**	
		
**N**	**Marker density (N**_e_**/M)**	**BayesB**	**G-BLUP**
2000	20	-0.024	0.005
	1	-0.027	-0.025
1000	20	0.005	-0.006
	1	0.014	0.005
500	20	-0.012	-0.014
	1	-0.023	0.019

The effect of even more distant relationships between training and evaluation individuals was investigated by continuing the breeding of the lines in Figure 1 for two more generations. This resulted in G4t and G4e individuals, which were separated by four more generations than the G2t and G2e individuals. Using density 20 N_e_/M and 2000 G4t individuals, the accuracy reduced to 0.920 and 0.868 for BayesB and G-BLUP respectively (result not shown elsewhere). These accuracies compare to those in Table 1, i.e. 0.928 and 0.881, respectively. Thus, the four additional generations of genetic drift, and thus change of LD, did not reduce the accuracies much, especially not for BayesB, which seemed to yield more persistent estimates of SNP effects over generations.

### Estimation of the effective number of loci

Using the results of Table 1 for a density of 20 N_e_/M, the regression of PEV^-1 ^on N was calculated as suggested in section 'Estimation of the effective number of segments'. For G-BLUP, the estimates of the intercept (α) and slope (β) were 1.388 and 1.551*10^-3^, respectively. For BayesB, these figures were 1.859 and 2.649*10^-3^, respectively. This results in estimates of the effective number of QTL of 516 and 302 for G-BLUP and BayesB, respectively. This value is expected to be lower for BayesB, since it concentrates on the loci with substantial effects whereas G-BLUP gives equal weight to all loci. The actual number of QTL was 120, which indicates that BayesB had to use several SNP to estimate the effect of each QTL. The derivation of Goddard [11] (see Section 'The scaling by N_e _argument') predicts that there are effectively 189 segments, which is considerably lower than the estimate of 516 by G-BLUP. Possibly the estimate of G-BLUP is biased by the deliberate exclusion of close relationships between the training and evaluation individuals. The estimates of the regression coefficients α and β can also be used to predict PEV, and thus r_G-BLUP _and r_BayesB _for different sizes of the training data set, N, than those used here.

Using the prediction of 189 effective segments from [11], Equation (1) predicts accuracies of 0.946, 0.899, and 0.824, for N = 2000, 1000 and 500, respectively. This is reasonably close to the BayesB accuracies, but should in fact be compared to the G-BLUP (which was assumed to derive the 189 effective segments) accuracies, which are substantially lower (Table 1; 20 N_e_/M results). This could be due to the training and estimation individuals being less related than when they were randomly sampled from the population.

### Lower heritability

The effect of a reduced heritability was tested using a heritability of 0.5 instead of 0.8. For N = 2000, this yielded accuracies of 0.789 and 0.859 for G-BLUP and BayesB, respectively (result not shown elsewhere). Equation (1) predicts that accuracy does not change if N*h^2 ^remains the same, which is approximately the case for N = 1000 and h^2 ^= 0.8, and yielded accuracies of 0.817 and 0.882 (Table 1). Thus, this prediction of Equation (1) seems to hold approximately, although the accuracy seems to decrease somewhat faster than predicted as h^2 ^reduces. The latter may be because Equation (1) predicts basically the accuracy of a single (effective) locus, whereas, if accuracy is high, all other loci are also predicted accurately. If heritability, and thus accuracy is reduced, the accuracy of the other loci reduces as well and the overall accuracy reduces more than expected from single locus predictions.

### The number of QTL and distribution of their effects

The number of simulated QTL was quite large: 24 per Morgan, i.e. 720 for a 30 Morgan genome. In addition, the effective size was quite small, such that the expected LD between the QTL is substantial, i.e. from [22]:

 where d is the distance between the QTL. This implies that the effect of the previous QTL in part carries over to the next, and thus that there are measurable QTL effects everywhere across the genome. Thus, the genetic model resembles that of the infinitesimal model, which assumes that infinitely many small QTL are smeared across the genome. Results from large-scale genome-wide association studies in humans support this genetic model with relatively small and many QTL [4].

This genetic model with many, small QTL will especially be a disadvantage for BayesB, which attempts to estimate the variance of individual QTL, whereas G-BLUP a priori assumes that every marker has equal variance. Therefore, the results in Table 1, show a smaller advantage for BayesB relative to G-BLUP than Meuwissen *et al*. [2] found, who simulated only ~5 QTL per Morgan. However, in general, BayesB has the advantage of using an informative priori distribution, which agrees well with the simulated distribution of QTL effects. Therefore, an alternative distribution for the QTL effects was also investigated, namely the normal distribution, which makes it harder for BayesB to detect and give extra weight to large QTL (since there are fewer). With N = 2000 and density 20 N_e_/M, the accuracy of selection reduced to 0.914, 0.916 and 0.879 for BayesB, MIXTURE and G-BLUP, respectively (result not shown elsewhere). Thus, the effect of normal vs. exponentially distributed QTL effects was small, but larger for BayesB than for G-BLUP as might be expected. Although the difference is small and may well be due to sampling, the MIXTURE model seems to yield the highest accuracy when QTL effects are normally distributed, which may be expected since it attempts to estimate the prior distribution from the data, and the normally distributed QTL effects may be more in accordance with the assumptions underlying the MIXTURE model.

### Requirements for high accuracy

The results presented here imply that the accurate prediction of breeding values of unrelated individuals requires a set of ~10*N_e_*L SNP markers and ~2*N_e_*M genotyped and phenotypes training individuals for the estimation of SNP effects. The former requirement is likely to be achieved in species where the genome sequence is available, but the latter will be challenging. If we accept accuracies of 0.7 – 0.8, ~1*N_e_*L training individuals is sufficient. For humans, this still implies ~350,000 training records, which makes the risk prediction for truly polygenic diseases and for unrelated individuals probably impossible unless genotyping and phenotyping for such diseases becomes very common in the future.

For cattle, 1N_e_L implies N = 30,000. Using Holstein dairy bulls, VanRaden *et al*. [23] found accuracies of 0.7–0.8 using N = 3,576, but in this situation the training and evaluation bulls were often highly related, and genomic EBVs were combined with T-BLUP EBVs, which were based on a much larger data set. Thus, the aforementioned requirements can be substantially reduced if the training and evaluation individuals are related, and Equation (2) can be used to predict by how much they can be reduced.

## Conclusion

1. Accuracies of ~0.9 for unrelated individuals require 10*Ne*L SNPs and 2*Ne*L training records. For related individuals these requirements can be substantially lowered.

2. Accuracy increases approximately linearly with marker density, when expressed as |R| between adjacent markers.

3. The superiority of BayesB over G-BLUP increases with marker density.

4. BayesB yielded more persistent estimates of SNP effects over generations.

5. As the size of the training data set increases, the difference between G-BLUP and BayesB decreases.

6. To take advantage of high marker densities, large training data sets are needed.

7. The regression of the inverse of the prediction error variance (PEV^-1^) on the number of training records (N) is linear, and the regression coefficients can be used to predict the accuracy for different N.

8. The scaling arguments predicted from Equation (1) hold approximately, but they over-predicted the accuracies found here, perhaps because the training and evaluation individuals were less related than expected.

## Competing interests

The author declares that they have no competing interests.

## Authors' contributions

THEM performed the computer simulations and wrote the manuscript.
